# In-flight transmission of wild-type SARS-CoV-2 and the outbreak potential of imported clusters of COVID-19: a review of published evidence

**DOI:** 10.1186/s12992-021-00749-6

**Published:** 2021-08-21

**Authors:** David Kelly, Niamh Bambury, Mairin Boland

**Affiliations:** 1 Department of Public Health East, Health Service Executive, Dublin, Ireland; 2 Health Protection Surveillance Centre, Dublin, Ireland

**Keywords:** Air travel, SARS-CoV-2, COVID-19, Port health, Quarantine

## Abstract

**Abstract:**

International air travel has been highlighted as a concern since the beginning of the COVID-19 pandemic with respect to importation of cases. We summarise the available evidence for in-flight transmission of wild type SARS-CoV-2 during 2020, and for imported COVID-19 clusters to cause outbreaks. This paper provides a data baseline prior to the emergence of new mutations causing SARS-CoV-2 variants of concern, whose characteristics may increase the potential risk of in-flight transmission and imported outbreaks. The evidence on in-flight transmission of wild-type SARS-CoV-2 is limited, and is described in a small number of published reports. Most of the available evidence pertains to the early phase of the COVID-19 pandemic, during a period without non-pharmaceutical interventions such as distancing and in-flight mask wearing. There is considerable potential for outbreaks of COVID-19 from imported cases or clusters when public health guidance around quarantine of travellers and self-isolation of cases is not adhered to. Risks can be mitigated by measures such as: avoiding non-essential travel, targeted testing and quarantine of travellers from high incidence regions or regions of concern, managed quarantine processes, and protocols for rapid investigation and control of transmission from a possible variant of concern. Measures should be dynamically assessed and proportionate to the level of risk.

**Graphical abstract:**

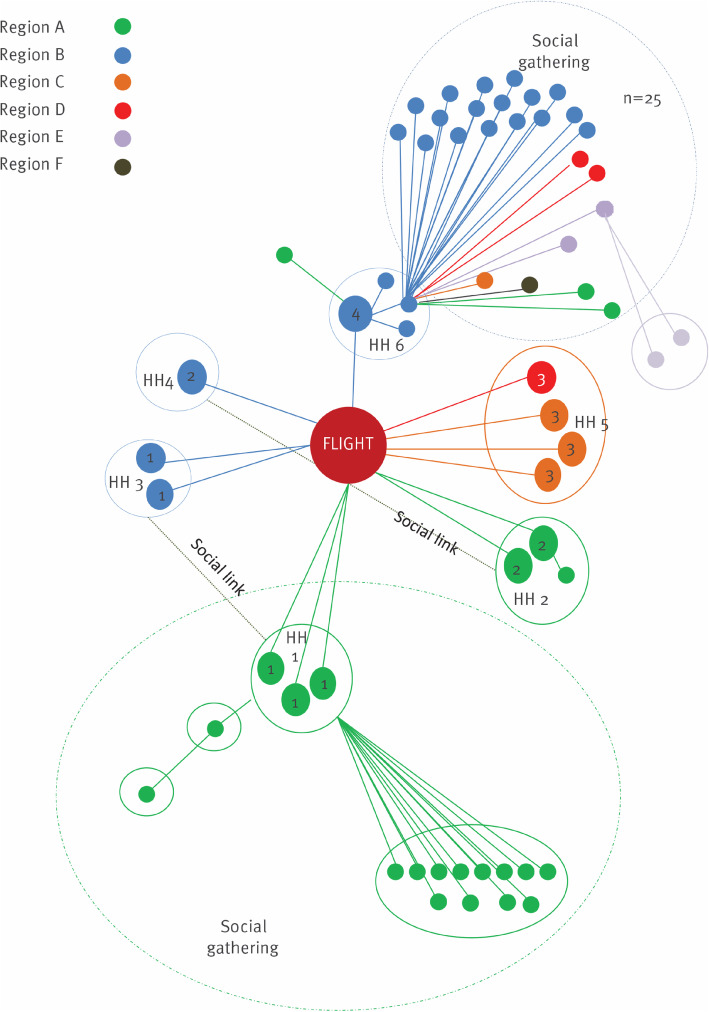

**Supplementary Information:**

The online version contains supplementary material available at 10.1186/s12992-021-00749-6.

## Background

Under International Health Regulations (IHR) [1], nations should provide a public health response to the international spread of disease in ways that are commensurate with public health risks, and which avoid unnecessary interference with international traffic and trade.

European Union guidance for a co-ordinated approach to travel restrictions through the COVID-19 pandemic aspires to an integrated method based on the epidemiological picture at a given time in the region of origin/destination [2], permitting member states to adopt their own health measures.

We review the available evidence on the risk posed by air travel during the first phases of the COVID-19 pandemic, when wild-type SARS-COV 2 predominated internationally. This provides a baseline for comparison as variants of concern (VOC) emerge. Published articles describe events; editorial commentary in academic journals draws on published evidence. Flights where no transmission occurs have not generally been reported upon, except in the early months of the pandemic, often on repatriation flights. This review can only provide a picture of events described and should be set in the context of substantial (albeit greatly reduced) numbers of international flights. Clearly the epidemiological picture of the country the passenger travels from is one key determinant of risk. To demonstrate the counterpoint: one study [3] described an outbreak of 59 cases of COVID-19 which stemmed from 13 flight cases linked by a 7 h, 17% occupancy flight to Ireland in summer 2020, with an attack rate of 17.8%; an aviation public health initiative published the same month determined that non-pharmaceutical interventions on commercial aircraft effectively dilute and remove pathogens, and in combination with face masks, results in a very low risk of SARS-COV-2 disease transmission on aircraft [4].

This literature review of in-flight transmission of wild-type SARS-CoV-2, and the potential for onward transmission, provides an important baseline of evidence, as VOC emerge internationally.

## Methods

A literature review was conducted on in-flight transmission of SARS-CoV-2, and on the outbreak potential of imported COVID-19 clusters from air travel. We aimed to present the available evidence on in-flight transmission events, the nature of infection prevention and control (IPC) measures and the risk of outbreaks of COVID-19 in the destination countries. Articles published from 1st January to 1st December 2020 were included. Inclusion criteria were articles with an index case or cases whose infectious period was during the flight and articles with identification of close contacts tested for COVID-19 within the 14-day incubation period following the flight. This resulted in a total of 19 articles [3, 5–22]. The denominator in calculated attack rates included the susceptible flight contacts, excluding those already confirmed as or infectious with COVID-19 at the time of the flight. Confirmatory whole genome sequencing (WGS) of SARS-CoV-2 isolates was reported by several studies, to confirm the relatedness of isolates and provide further evidence of secondary transmission by pairwise comparison of nucleotide sequences between the in-flight cases and contacts.

### Findings

Of the 19 articles reviewed, 11 reported possible evidence of in-flight transmission of SARS-CoV-2 (see supplementary data in Table 1) [3, 5–10, 12–14, 16]. The calculated attack rates ranged from 0 to 6.9% among the exposed passenger and cabin crew, which included both close and casual contacts. The highest attack rates were reported on flights from Sydney to Perth (4.9%) and from UK to Vietnam (6.9%), both in March 2020 [5, 7]. Face masks were not mandatory at the time, and reportedly worn by few passengers on the Australian flight. For the flight from UK to Vietnam, the article did not report on mask wearing. There was evidence of transmission from passengers to crew members in two articles [6, 7]. Both flights involved business class travel in March 2020, and neither article reported on mask wearing by passengers.

One review article on the evidence of in-flight transmission of SARS-CoV-2 and attack rates deemed in-flight transmission epidemiologically highly likely [22]. All three flights reviewed involved flight durations over five hours, without mandatory mask wearing, during March 2020 [5–7]. The calculated attack rates for these flights were 0.7, 4.9 and 6.9%. Evidence of in-flight transmission was proven by whole genome sequencing of SARS-CoV-2 isolates in two of these articles [5, 6].

Whole genome sequencing can contribute further evidence on flight-related transmission. A study [3] of 13 cases linked by a 7 h, 17% occupancy flight, with pairwise comparison of the nucleotide sequences of five samples showed more than 99% homology across the entire viral genome, strongly suggesting a single point source of infection. The plausible calculated flight attack rate was 17.8%, (minimum 9.8%; maximum 25%) [3].

Absence of in-flight transmission of SARS-CoV-2 was evidenced by two articles [11, 15]. A 15h flight from China to Canada of 350 passengers had two cases of COVID-19 on board in January 2020. Of the 25 close contacts actively monitored for 14 days, one later became symptomatic and tested negative for SARS-CoV-2. Five other passengers became symptomatic and also tested negative for SARS-CoV-2; an attack rate of zero [15]. Mask wearing was reported on this flight, though not quantified. A 14 h evacuation flight of 11 passengers from Japan to Israel had two cases of COVID-19 on board in February 2020. The nine remaining passengers were repeatedly tested for SARS-CoV-2 during the 14 day quarantine period after arrival. None tested positive for COVID-19 [11]. All passengers and crew wore either filtering face piece 2 (FFP2) or surgical face masks for the flight duration, on a small charter aircraft.

Low attack rates (< 1%) were identified on several flights [8, 16]. Two evacuation flights of 11 h duration from Italy to South Korea reported very low attack rates of only 0.3% (1/293) and 0.5% (1/202) among exposed passengers. FFP2 masks were reportedly worn by most passengers, who were all tested on day 1 and day 14 of arrival [8]. Three evacuation flights from China to Japan of six hours duration had in total eight cases of COVID-19 on board [16]. All passengers and crew were quarantined at hotel accommodation for 12 days and tested for SARS-CoV-2 on arrival and again on day 13. Calculated attack rates were 0.5% (1/202), 0.5% (1/208) and 0.7% (1/148) for the flights. The article did not report on the wearing of face masks by passengers or crew on these flights in January 2020. A 10 h flight from China to Singapore in January 2020 reported an attack rate of 1.1% (1/92) among exposed passengers [14]. Surgical masks were provided to all passengers on the plane; the wearing of these was not quantified. All passengers underwent mandatory 14 day quarantine at a government facility on arrival, and were tested for SARS-CoV-2 on day 6.

Where reported, mask wearing appeared to reduce the attack rate of COVID-19 on aircraft, potentially to zero when correctly adhered to [11, 14, 15]. There is limited evidence of SARS-CoV-2 transmission from passengers to crew members on aircraft, outside of business class. In-flight transmission, where purported to have occurred, mostly involved contacts seated in proximity to index cases, at an early phase of the COVID-19 pandemic before April 2020.

The outbreak potential for imported clusters of COVID-19 was reported in many articles (see supplementary data in Table 2) [3, 13, 17–21]. All involved at least two index cases (range 3 to 48) with a history of travel from countries of high COVID-19 incidence, to countries of lower COVID-19 incidence, often within an organised tour group who shared transport, accommodation and meals. Attack rates among the tour groups and any potential spread beyond, along with any quarantine measures implemented upon return from travel are described. The reported attack rates ranged from 8.2 to 90.5%. Four of the reviewed articles reported outbreak spread beyond the initial tour group clusters; none featured mandatory quarantine of cases or close contacts [3, 13, 18, 19]. Two of these imported clusters resulted in national outbreaks [3, 18].

## Discussion

This literature review found fewer than twenty articles demonstrating in-flight transmission of SARS-CoV-2 in 2020. There was clear but limited evidence of events of in-flight transmission, proven in some cases by whole genome sequencing.

Studies of high-occupancy flights from areas of high COVID-19 incidence, with infectious case(s) on the aircraft, suggest however that transmission may be reduced when in-flight IPC measures are implemented and adhered to. Most studies [5–7, 22] involved flights taken prior to April 2020, when limited IPC measures such as social distancing and mask wearing were in place [3, 5–7, 22], with one occurring in summer 2020. It was also evident from the published articles that IPC measures varied in their implementation and passenger adherence, as did reporting of the presence or absence of IPC measures. Where in-flight IPC measures such as the wearing of face masks are adhered to, low attack rates are generally demonstrated [8, 11, 14–16].

Where in-flight transmission from passengers to crew was reported, the business class setting was identified in both instances. Business class is available on longer flight durations, with prolonged exposure and increased face-face interaction with crew from serving of food and beverages. Crew may also share in-flight toilet facilities within business class as opposed to economy class. Business class passengers may also remove face masks for a longer cumulative duration in business class owing to increased servings of food and beverages. Aircraft operators are now advised by guidance to limit passenger crew interaction in all sections of the plane [23].

The outbreak potential for imported clusters found that higher attack rates of COVID-19 were reported among tour groups (8–91%) than among aircraft passenger contacts (< 7%). This is consistent with household contact being the highest risk for transmission of COVID-19, as tour groups spend prolonged time sharing meals, transport and accommodation. One large imported COVID-19 cluster resulted in secondary and tertiary transmission to household contacts, and social contacts [3] with suboptimal compliance regarding isolation and restriction of movements for cases and contacts respectively.

A recent evidence synthesis demonstrated a wide variation in the period of restricted movement posttravel across Europe, ranging from 7 to 14 days with and without testing [24]. An evidence summary demonstrated that a 14 day period of restricted movement captures approximately 95% of individuals who will become symptomatic [25]. Reducing this to ten or seven days would capture approximately 84 and 64% of individuals, respectively. This would potentially have a significant impact on numbers of secondary and tertiary cases associated with imported cases of COVID-19.

The World Health Organisation (WHO) stated in December 2020 that international travellers should not be considered by nature as suspected COVID-19 cases or contacts, and did not recommend travellers as a priority group for testing [26]. As novel SARS-CoV-2 VOC emerge, targeted sequencing of isolates from travellers from high risk countries, in parallel with ongoing community sampling may allow for detection of imported VOC and the extent of their local transmission. The increased transmissibility of VOC has led to introduction of more stringent control measures against importation of cases/clusters, given their potential for outbreaks, and increased hospitalisations, morbidity and mortality [27]. Such measures include a risk-based approach to escalation of travel restrictions in compliance with Article 43 of IHR [28]. The European Centre for Disease Prevention and Control (ECDC) risk assessment updated in February 2021 advises against non-essential travel. In addition, increased testing and quarantine measures for travellers is recommended, in particular those from areas with a higher incidence of VOC [29].

Mandatory managed quarantine of returning travellers from regions of high COVID-19 incidence has been used to limit the outbreak potential of imported clusters in reviewed articles [14, 17, 20, 21]. The European Commission has recently recommended a common approach towards targeted isolation of COVID-19 patients and quarantine for contacts and travellers, with rigorous contact tracing [30]. Member States may require persons travelling from an area of another Member State that is classified other than ‘green’ to undergo quarantine; and/or undergo a test for COVID-19 infection after arrival [2].

As for a dynamic risk assessment of travel, the ‘Public Health corridor’ concept has been introduced by CAPSCA [31]; this is a multi-layered approach to mitigate risks of air travel and to assess inter country risks. WHO has recently proposed a risk assessment tool to inform proportionate mitigation measures for international travel [32]. Since July 2021, free movement of air travel passengers among EU member states has been facilitated by implementation of the EU Digital COVID Certificate (DCC) [33]. The DCC requires that airline passengers provide proof of either immunity to COVID-19 from previous infection, COVID-19 vaccination, or a negative PCR test for SARS-CoV-2 taken within 72 h or negative antigen test taken within 48 h pre-departure. This effectively reduces the population susceptible to in-flight transmission of SARS-CoV-2 on board aircraft to those solely with a negative pre-departure test.

Since publication of the reviewed studies, widespread transmission of the SARS-CoV-2 Delta (B.1.617.2) variant has occurred, which now constitutes the dominant strain of SARS-CoV-2 circulating in the majority of countries. The Delta variant has been linked to increased transmissibility and severity of COVID-19 infection [34], which may result in increased in-flight transmission from an index case.

## Conclusion

A small number of studies have shown evidence of in-flight transmission of SARS-CoV-2 in the early phase of the COVID-19 pandemic, with higher attack rates associated with tour groups and with business class settings; and lower attack rates associated with adherence to in-flight mask wearing.

As the world grapples with the Delta VOC, amid drives to vaccinate citizens, it is clear that sustained and strengthened public health measures are needed. With increasing vaccine coverage globally, the population susceptible to in-flight transmission will diminish further, which will likely counterbalance and may eventually outweigh the increased transmissibility of the Delta variant, reducing attack rates to very low levels.

We need dynamic evidence-based determinations on the risk, or lack of added risk, posed by travel from other countries. Key public health measures include not travelling while symptomatic, social distancing, cleaning and hygiene measures, reduced interaction between passengers and crew, consideration of pre-travel testing and scheduled testing on arrival, and adherence to quarantine, which is becoming mandatory in some EU member states. Prompt isolation of cases and robust contact tracing remain key to preventing onwards transmission [35].

## Supplementary Information

**Additional file 1: Table 1.** Reviewed articles on evidence of in-flight transmission of SARS-CoV-2.

**Additional file 2: Table 2.** Reviewed articles on outbreak potential of imported clusters of COVID-19.

## Data Availability

All data generated or analysed during this study are included in this published article [and its supplementary information files].

## Abbreviations

DCC: Digital COVID Certificate
EU: European Union
ECDC: European Centre for Disease Prevention and Control
FFP2: Filtering Face Piece 2
IHR: International Health Regulations
IPC: Infection Prevention and Control
PCR: Polymerase Chain Reaction
VOC: Variants of Concern
WHO: World Health Organisation
