# Supplementary material for: In-flight transmission of wild-type SARS-CoV-2 and the outbreak potential of imported clusters of COVID-19: a review of published evidence
Source: Global Health. 2021 Aug 21;17:93. doi: 10.1186/s12992-021-00749-6 (PMC8379567; doi:10.1186/s12992-021-00749-6)
Supplement: Supplementary file 1 — Additional file 1: Table 1. Reviewed articles on evidence of in-flight transmission of SARS-CoV-2. [file 12992_2021_749_MOESM1_ESM.docx]

**SUPPLEMENTARY MATERIAL**

**Table 1: Reviewed articles on evidence of in-flight transmission of SARS-CoV-2**

| **Title** | **Flight duration** | **Origin** | **Destination** | **Cases on board** | **Exposed Contacts** | **Attack rate** | **Quarantine** | **Findings incl. mask wearing** |
| --- | --- | --- | --- | --- | --- | --- | --- | --- |
| In-flight Transmission SARS-CoV-2: review of attack rates and available data on the efficacy of face masks | Review article | Review article | Review article | Review article | Review article | Review article | None mandatory | 3 flights highly likely aircraft transmission. 2 proven by WGS. Lack of mask wearing where transmission. Cases in proximity to index. |
| A large national outbreak of COVID-19 linked to air travel, Ireland, summer 2020 | 7h | Qatar | Ireland | 3 | 45 | 17.8%  (8/45) | None. Symptomatic and asymptomatic tested. | Mask wearing variable among cases. Unknown among contacts |
| Flight-Associated Transmission of Severe Acute Respiratory Syndrome Coronavirus 2 Corroborated by Whole-Genome Sequencing | 5h | Sydney | Perth | 9 | 223 | 4.9%  (11/223) | Quarantine advised. Only symptomatic tested. | 9/18 primary cases infectious on flight. Masks worn rarely. WGS proven transmission |
| In-Flight Transmission of SARS-CoV-2 | 15h | USA | Hong Kong | 2 | 294 | 0.7%  (2/292) | None. Only close contacts tested | Masks optional. Both secondary cases cabin crew. WGS proven |
| Transmission of SARS-CoV 2 During Long-Haul Flight | 10h | UK | Vietnam | 1 | 216 | 6.9%  (15/216) | Home quarantine. Symptomatic and asymptomatic tested | Optional masks.  Secondary cases in proximity to index |
| Asymptomatic Transmission of SARS-CoV-2 on Evacuation Flight | 11h  11h | Italy | South Korea | 6  3 | 293  202 | 0.34% (1/293)  0.50% (1/202) | Quarantine in place on arrival | Case wore mask. Seated close to index. No crew cases. 1 passenger. |
| Assessment of SARS-CoV-2 Transmission on an International Flight and Among a Tourist Group | 5h | Israel | Germany | 7 | 102 | 2.1% (2/95) | Asymptomatic and symptomatic tested. Quarantine | No masks worn.  2 cases closely seated to index |
| SARS-CoV-2 Screening Test for Japanese Returnees From Wuhan, China, January 2020 | 6h  6h  6h | China | Japan | 4  2  2 | 202  208  148 | 0.5%  (1/202)  0.5%  (1/208)  0.7%  (1/148) | 3 evacuation flights.  All tested day0 and day13.  All quarantined. | No report of masks worn in article |
| In-flight transmission cluster of COVID-19: a retrospective case series | 5h | Singapore | China | 1 | 325 | 3.4% (11/324) | None. Quarantine on arrival. Onset >3 days | No mask worn.  Cases all passenger  Crew wore masks |
| Absence of in-flight transmission of SARS-CoV-2 likely due to use of face masks on board | 14h | Japan | Israel | 2 | 9 | 0%  (0/9) | None. All tested.  Quarantine on arrival | FFP2 masks worn |
| Potential transmission of SARS-CoV-2 on a flight from Singapore to Hanghzou, China: An epidemiological investigation | 5h | Singapore | China | 3 | 332 | 3.9% (13/332) | Quarantine on arrival. 3 tour groups. | 1 passenger case  No crew cases  Case wore a mask - removed |
| Lack of COVID-19 transmission on an international flight | 15h | China | Canada | 2 | 350 | 0%  (0/350) | Symptomatic only.  6/6 tested negative | Close contacts active monitoring No masks worn |
| Probable aircraft transmission of Covid-19 in-flight from the Central African Republic to France | 2h | CAR | Cameroun | 1 | N/A | Unknown | Transmission  possibly from partner | Partner was clinical diagnosis. But tested negative day8. |
| SARS-CoV-2 Infection among Travelers Returning from Wuhan, China | 10h | China | Singapore | 2 | 92 | 1.1% (1/92) | Quarantined 14day  All tested | Surgical masks worn |
