# Supplementary material for: In-flight transmission of wild-type SARS-CoV-2 and the outbreak potential of imported clusters of COVID-19: a review of published evidence
Source: Global Health. 2021 Aug 21;17:93. doi: 10.1186/s12992-021-00749-6 (PMC8379567; doi:10.1186/s12992-021-00749-6)
Supplement: Supplementary file 2 — Additional file 2: Table 2. Reviewed articles on outbreak potential of imported clusters of COVID-19. [file 12992_2021_749_MOESM2_ESM.docx]

**SUPPLEMENTARY MATERIAL**

**Table 2: Reviewed articles on outbreak potential of imported clusters of COVID-19**

| **Title** | **Origin** | **Destination** | **Cases** | **Contacts** | **Attack rate** | **Containment** | **Findings** |
| --- | --- | --- | --- | --- | --- | --- | --- |
| A large national outbreak of COVID-19 linked to air travel, Ireland, summer 2020 | Qatar | Ireland | 13 | N/A | N/A | No. 46 secondary cases in national outbreak.  No quarantine | Symptomatic and asymptomatic tested. |
| Importing coronavirus disease 2019 (COVID-19) into China after international air travel | Singapore | China | 10 | 110 | 8.2%  (9/110) | Yes. All flight quarantined in China | Tested symptomatic only. Tour group=110.  Possible aircraft transmission of 1 case |
| Clusters of 2019 coronavirus disease (COVID‐19) cases in Chinese tour groups | China | Italy | 13 | 34 | 38.2%  (13/34) | Yes. Group quarantined on return to China | 6/13 radiological, not laboratory confirmed |
| A cluster of SARS-CoV-2 infection among Italian tourists visiting India, March 2020 | Italy | India | 17 | 26 | 65.4% (17/26) | Unknown. Not discussed. | Asymptomatic and symptomatic tested |
| A cluster of COVID-19 in pilgrims to Israel | Israel | Greece | 48 | 53 | 90.5%  (48/53) | No, 5 cases among close contacts aircraft | Asymptomatic and symptomatic tested |
| Assessment of SARS-CoV-2 Transmission on an International Flight and Among a Tourist Group | Israel | Germany | 7 | 24 | 29.2%  (7/24) | No. 2 probable cases aircraft transmission.  No quarantine | Asymptomatic and symptomatic tested. |
| Emergence of coronavirus disease 2019 (COVID-19) in Austria | Italy | Austria | 3 | N/A | N/A | No. Outbreak of 23 additional cases | Ischgl ski resort outbreak. Not possible to determine attack rate |
